# Social participation is an important health behaviour for health and quality of life among chronically ill older Chinese people

**DOI:** 10.1186/s12877-020-01713-6

**Published:** 2020-08-24

**Authors:** Zeyun Feng, Jane Murray Cramm, Anna Petra Nieboer

**Affiliations:** 1 Department of Socio-Medical Sciences, Erasmus School of Health Policy & Management, Erasmus University Rotterdam, P.O. Box 1738, DR. Rotterdam, Rotterdam, 3000 the Netherlands; 2 Department of Health Technology Assessment, Shanghai Health Development Research Center (Shanghai Medical Information Center), Jianguo Road 602, Shanghai, 200031 China

**Keywords:** Social participation, Health outcome, Health behaviour, Older adult, Quality of life, Chronic disease

## Abstract

**Background:**

Health behaviours (physical activity, maintenance of a healthy diet and not smoking) are known to be beneficial to the health and well-being of chronically ill people. With China’s ageing population and increased prevalence of people with chronic diseases, the improvement of unhealthy behaviours in this population has become crucial. Although recent studies have highlighted the importance of social participation for health and quality of life (QoL) among older people, no study to date has included social participation along with more traditional health behaviours. Therefore, this study aimed to identify associations of multiple health behaviours (social participation, physical activity, maintenance of a healthy diet and not smoking) with health and QoL outcomes (including cognitive and physical function) among chronically ill older adults in China.

**Methods:**

For this nationally representative cross-sectional study, wave 1 data from the World Health Organization’s Study on global AGEing and adult health (China) were examined. In total, 6629 community-dwelling older adults (mean age, 64.9 years) with at least one chronic disease were included. Multivariate linear regression analyses were used to evaluate associations of health behaviours with health and QoL outcomes while controlling for background characteristics.

**Results:**

Greater social participation was associated with better QoL [β = 0.127, standard error (SE) = 0.002, *p* < 0.001], cognitive function (β = 0.154, SE = 0.033, *p* < 0.001) and physical function (β = − 0.102, SE = 0.008, *p* < 0.001). Physical activity was associated with better QoL (β = 0.091, SE = 0.015, *p* < 0.001) and physical function (β = − 0.155, SE = 0.062, *p* < 0.001). Sufficient fruit and vegetable consumption was associated with better QoL (β = 0.087, SE = 0.015, *p* < 0.001).

**Conclusions:**

Our findings suggest that social participation is an important health behaviour for quality of life and cognitive function among chronically ill older people in China. Health promotion programmes should expand their focus to include social participation as a health behaviour, in addition to physical activity, maintenance of a healthy diet and not smoking.

## Background

Humans are living much longer today than they did 100 years ago; this great achievement in human development is accompanied by new challenges [1]. Chronic diseases pose an increasing global problem [2], and older adults are more vulnerable to such conditions (e.g. cardiovascular diseases, diabetes and lung diseases) [3].

China has the largest ageing population in the world, and the rate of ageing in this country has accelerated over recent years [4]. At the end of 2018, the population of China included more than 249.49 million (about 17.9%) people aged ≥60 years [5]. Approximately 150 million of these older adults have at least one chronic illness [6]. For decades, research has consistently shown that people with chronic conditions are at greater risk of worse quality of life [7–9] and health outcomes [10] than are those without chronic disease. Thus, the identification of modifiable factors to prevent the deterioration of health and quality of life among chronically ill older adults is crucial in a time of ageing societies.

Considerable evidence shows that healthy lifestyle habits, such as physical activity and maintenance of a healthy diet, can slow the deterioration of cognitive function, quality of life and physical function in chronically ill (older) populations [11–16]. For example, physical activity has been associated with better cognitive function among older adults with hypertension [16], and has been found to enhance the quality of life of patients with type 2 diabetes [12–15] and heart failure [11, 12].

Not only traditional health behaviours (i.e. physical activity, maintenance of a healthy diet and not smoking), but also older people’s ability to stay socially active and connected to others is essential for health and quality of life outcomes. Social participation is considered to be a critical element of active ageing [17] and has been incorporated into many theoretical models of successful ageing [18]. It has been associated with longevity [19], self-rated health [6], quality of life [20, 21] and functional ability [22]. Notably, the positive influence of social participation on health was found to be greatest among older adults [23]. For example, the association between social participation and cognitive function was shown to be stronger among older adults than among younger persons [22]. A possible explanation is that active engagement in social activities gives older people opportunities to experience more dynamic environments, which is considered to be beneficial for the maintenance of cognition by stimulating neurogenesis, even at older ages [22].

Less attention has been paid to whether chronically ill older adults can benefit from social participation [24, 25]. Several studies have shown that social participation affects the (health-related) quality of life of older adults with arthritis [26, 27] and post-stroke [28]. Research on chronically ill older Chinese adults, however, is limited. In the first study of its kind, Hu and colleagues [29] found no association between social participation and quality of life among older Chinese adults with diabetes. However, their measurement of social participation focused mainly on formal organisations (e.g. sports clubs), which might have led to underestimation and contributed to inaccurate estimation of this association; in China, joining formal social organisations, such as sports clubs and culture associations, is not common [29], whereas activities such as public square dancing (guang chang wu in Mandarin) [30], group tai chi practice [31] and group singing in parks [32] are common. Furthermore, Hu and colleagues’ [29] findings were not generalisable to the whole country because of the sampling strategy used.

More importantly, although previous research has identified the importance of traditional health behaviours and social participation separately, no study to date has incorporated social participation as a health behaviour in addition to physical activity, maintenance of a healthy diet and not smoking. Thus, the purpose of this study was to investigate the associations of social participation and these traditional health behaviours with health and quality-of-life outcomes among chronically ill older adults in China, using a large nationally representative dataset.

## Methods

### Participants and data

Data for this study were taken from wave 1 of the World Health Organization’s (WHO’s) Study on global AGEing and adult health (SAGE), the most recent data available from China. SAGE is a longitudinal study for which nationally representative data were collected from adults aged ≥50 years from six low- and middle-income countries (China, Ghana, India, Mexico, the Russian Federation and South Africa) using a multistage, stratified cluster sampling approach. The effectiveness and high response rate of SAGE are attributable to proper planning and organization from the initiation of the study [33]. All investigators, supervisors and interviewers were trained to administer the survey in the field, introduce SAGE to the sampled households [34]. In China, wave 1 of SAGE was implemented in 16 strata in 8 provinces/municipalities [34]. A five-stage cluster sampling strategy was used to select participants, who were contacted by telephone or in person, and about 200 investigators were involved in wave 1 data collection via face-to-face interviews between 2008 and 2010 [34]. About half of the interviews were computer assisted (CAPI), and half involved manual data recording [35]. Investigators visited the selected households and collected information about household rosters; then, the survey team completed the questionnaires at a central location (e.g. a neighbourhood office) or at respondents’ homes [34]. Each respondent received a small gift for his or her cooperation [34]. An excellent response rate was achieved (93%), similar to rates for other surveys (e.g. the China Health and Retirement Longitudinal Study) conducted among older people in China. Detailed information about the SAGE data collection procedures can be found elsewhere [34].

**Fig. 1:**
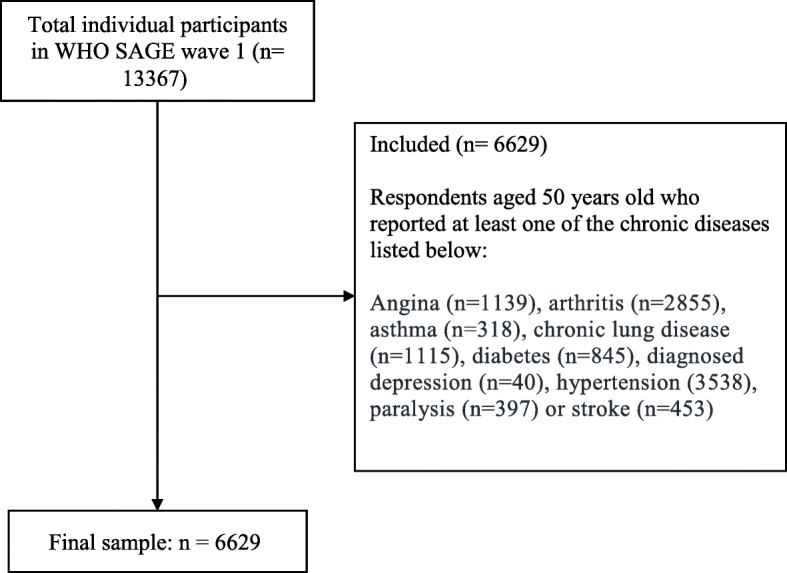
Flow chart on how the final sample (*n* = 6629) was derived

SAGE consists of national longitudinal studies of older people (age ≥ 50 years) in six lower- and upper–middle-income countries. The instruments and threshold age used are compatible with other large longitudinal ageing studies conducted in high-income countries, such as the US Health and Retirement Study (HRS) and the Korean Longitudinal Study on Ageing (KLoSA), enabling sound international comparisons of the ageing process, health and well-being among middle-aged and older adults [35]. The original wave 1 sample included 13,367 participants from China. We enrolled respondents aged ≥50 years with chronic disease (angina, arthritis, asthma, chronic lung disease, diabetes, diagnosed depression, hypertension, paralysis or stroke), leading to a final sample of 6629 respondents. Most (*n* = 6194, 93.4%) older persons in the sample were aged 50–80 years; people aged 50–59 years made up the largest group (*n* = 2270, 34.2%), those aged 60–69 years comprised the second largest group (*n* = 2154, 32.5%) and only 6.6% (*n* = 435) of the sample was aged > 80 years. The procedure for sample selection is summarized in Fig. 1.

### Measures

#### Chronic conditions

For self-reporting of chronic conditions, respondents were asked whether they had been diagnosed with any of the following: i) angina or angina pectoris (heart disease), ii) arthritis (or rheumatism, osteoarthritis), iii) asthma (an allergic respiratory disease), iv) chronic lung disease (emphysema, bronchitis, COPD), v) diabetes (high blood sugar), vi) depression, vii) high blood pressure (hypertension), viii) paralysis and ix) stroke. The questions were formatted as: “Have you ever been diagnosed with/told by a health care professional you have …? ” Respondents provided yes/no answers. They were considered to have chronic (a) disease(s) if they answered “yes” to any of the questions.

#### Health behaviours

Social participation was measured using summed scores for the 9-item questionnaire developed for the SAGE [36] (Additional file 1). Items enquire about respondents’ frequency of community involvement in the past 12 months, with responses ranging from ‘never’ (1) to ‘daily’ (5). The Cronbach’s alpha value for the questionnaire in this study was 0.63. We used adequate fruit and vegetable intake as an indicator of healthy diet (insufficient, fewer than two servings of fruit and three servings of vegetables/day; sufficient, two or more servings of fruit and three or more servings of vegetables/day] [37]. Version 2 of the General Physical Activity Questionnaire was used to measure physical activity [36]. Participants were asked to report the average number of days per week and time in which they engaged in vigorous and moderate physical activity. We recorded physical activity as sufficient or insufficient according to the WHO threshold of 150 min/week [38]. Smoking habits were assessed by asking whether participants were daily smokers (yes/no).

### Outcome variables

#### Quality of life

Quality of life was measured using the 8-item World Health Organization quality of life measure (WHOQoL) [35] (Additional file 2). Respondents were asked to rate their satisfaction with life in general and in different domains (e.g. finances, health and relationships) on a 5-point scale ranging from 0 (‘not at all/very poor’) to 5 (‘completely/very good’). Total scores were calculated by summing the item scores and rescaling the result to 0–100 [39]. According to previous research [40], the 8-item WHOQoL is useful for the assessment of quality of life in older populations. The Cronbach’s alpha value of the instrument in this study was 0.86.

#### Cognitive function

Cognitive function was measured by administering five cognitive performance tests (forward and backward digit spans, immediate and delayed verbal recall, and verbal fluency) [41]. Forward digit span was tested by asking participants to repeat progressively longer number series in the exact order in which they had been presented [41, 42]. Backward digit span was tested by asking participants to repeat such series backwards [41]. Scores (longest spans repeated) for the forward and backward digit spans ranged from 0 to 9 and 0 to 8, respectively (total possible scores, 1–17) [42]. Immediate and delayed verbal recall was measured by asking participants to read 10 words aloud and soon thereafter to recall as many words as possible in 1 min [41]. The same test was repeated three times. Scores ranged from 0 to 10 [43]. Verbal fluency was assessed by asking respondents to name as many animals as they could in 1 min [42]. Scores were based on the number of correctly named animals, with repeated names counted only once (range, 2–38) [42, 43]. *Z* scores were calculated for the five test scores, and final cognitive function scores (range, 0–100) were generated by summing these scores [41, 42].

#### Physical function

Physical function was measured using the activities of daily living items from version 2 of the WHO’s Disability Assessment Schedule, based on the Katz Index of Independence in Activities of Daily Living [44]. Total scores was calculated by summing scores for the following items: 1) difficulty in bathing/washing your whole body, 2) difficulty in getting dressed, 3) difficulty with getting to and using the toilet, 4) difficulty with standing up from sitting down, 5) difficulty in getting up from lying down and 6) difficulty with eating (including cutting up your food). Responses are structured by a 5-point scale ranging from 0 (none) to 4 (extreme/cannot do). The Cronbach’s alpha value for this instrument in this study was 0.89.

### Potential confounders

Based on data from the literature and the availability of SAGE data, we included age (in years), gender (male/female), marital status, area of residence (urban/rural), educational level and income (by quintile) as potential confounders because they are associated with both health behaviours and health outcome variables [45–51].

We dichotomized marital status as non-single (including ‘currently married’ and ‘cohabiting’) and single (including ‘never married’, ‘separated/divorced’ and ‘widowed’), and educational level as higher (completion of secondary school or more) and lower (completion of primary school or less). The Chinese government’s administrative division was used to determine if people lived in a rural or urban area. Respondents’ incomes were estimated. SAGE-China used the WHO’s Bayesian post-estimation method to generate raw continuous income estimates based on income indicators such as a set of household ownership of durable goods (e.g. number of chairs), various dwelling characteristics (e.g. type of floor) and access to services (improved water, sanitation and cooking fuel) [52, 53]. Estimated income was then transformed into quintiles [53], with quintile 1 denoting the lowest and quintile 5 denoting the highest income [52, 53].

### Statistical analysis

Descriptive statistics and frequencies were used to describe the study population. Correlation analysis was performed to assess relationships between background characteristics and health behaviours using the outcome measures (quality of life, cognitive function and physical function). Multivariate linear regression analyses were conducted to study associations between health behaviours (physical activity, maintenance of a healthy diet, smoking and social participation) and quality of life and health outcomes while controlling for background characteristics. We used listwise deletion of missing cases in the multivariate analyses. Analyses were performed using IBM SPSS software (version 24; IBM Corporation, Armonk, NY, USA). As the sample was large, the significance level was set at *p* < 0.001. All statistical tests were two sided.

## Results

### Participants’ characteristics

In total, 6629 participants with a mean age of 64.9 (range, 50–99) years were included in the study (Table 1). More than half (56.0%) of the participants were women. The majority of participants were non-single (81.9%) and had lower educational levels (60.4%). Fewer than half (42.9%) lived in rural areas. About one-fifth (20.5%) of the respondents were daily smokers, and more than one-third reported inadequate fruit and vegetable consumption and/or insufficient physical activity. The mean social participation index score was 14.6 (standard deviation, 3.58; range, 8–36). The percentages of missing values for the study variables were ≤ 7.1%.

**Table 1:** Characteristics of the study population (*n* = 6629)

Characteristic	*n*	%	Mean (SD)	Range
Age (years)	6629	100.0	64.9 (9.28)	50–99
Gender				
Female	3709	56.0		
Male	2920	44.0		
Marital status *Missing 6 (0.1%)*				
Non-single	5426	81.9		
Single	1197	18.0		
Residence				
Rural	2846	42.9		
Urban	3783	57.1		
Education level *Missing 35* (0.5%)				
Lower	3984	60.1		
Higher	2610	39.4		
Income level M*issing 30* (0.5%)				
Quintile 1 (lowest)	1265	19.1		
Quintile 2	1246	18.8		
Quintile 3	1333	20.1		
Quintile 4	1417	21.3		
Quintile 5 (highest)	1338	20.2		
NCDs				
Hypertension	3538	53.8		
Arthritis	2855	43.1		
Angina	1139	17.2		
Chronic lung disease	1115	16.9		
Diabetes	845	12.8		
Stroke	453	6.8		
Paralysis	397	6.2		
Asthma	318	4.8		
Depression diagnosed	40	0.6		
Health behaviours				
Social participation index *Missing 11 (0.2%)*	6618	99.8	14.6 (3.58)	8–36
FV consumption *Missing 333 (5%)*				
Inadequate	2188	33.0		
Adequate	4108	62.0		
PA M*issing 13 (0.2%)*				
Insufficient	2309	34.8		
Sufficient	4307	65.0		
Daily smoker M*issing 16 (0.2%)*				
Yes	1358	20.5		
No	5255	79.3		
Health and QoL outcomes				
QoL *Missing 121 (1.8%)*	6508	98.2	3.5 (0.6)	1–5
Cognitive function *Missing 471 (7.1%)*	6158	92.9	38.9 (10.1)	3–94
Physical function *Missing 15 (0.2%)*	6614	99.8	0.9 (2.4)	0–24

*SD* standard deviation; NCD, non-communicable disease; FV, fruit and vegetable; PA, physical activity; QoL, quality of life

### Correlations

Social participation showed weak positive correlations with quality of life (*r* = 0.178, *p* < 0.001) and cognitive function (*r* = 0.197, *p* < 0.001) scores, and a weak negative correlation with the physical function score (*r* = − 0.135, *p* < 0.001), indicating that greater degrees of social participation correlated with better quality of life, cognitive function and physical function (Table 2). Adequate fruit and vegetable intake showed weak positive correlations with quality of life (*r* = 0.185, *p* < 0.001) and cognitive function (*r* = 0.153, *p* < 0.001) scores, and a weak negative correlation with the physical function score (*r* = − 0.073, *p* < 0.001), indicating that it correlated with better quality of life, cognitive function and physical function (Table 2). Physical activity showed weak positive correlations with quality of life (*r* = 0.095, *p* < 0.001) and cognitive function (*r* = 0.105, *p* < 0.001) scores, and a weak negative correlation with the physical function score (*r* = − 0.197, *p* < 0.001), indicating that physically active individuals had better quality of life, cognitive function and physical function (Table 2). Daily smoking did not correlate with quality of life or cognitive or physical function (Table 2).

**Table 2:** Associations of background characteristics and health behaviours with quality of life and health outcomes

	QoL^a^	Cognitive function^b^	Physical function^c^
Age (years)	−.054***	−.310***	.228***
Gender (female)	−.055***	−.088***	.020
Residence (rural)	−.124***	−.256***	.119***
Marital status (non-single)	.122***	.189***	−.108***
Education (lower)	−.180***	−.374***	.130***
Income			
Quintile 1 (lowest)	−.252***	−.254***	.120***
Quintile 2	−.108***	−.143***	.021
Quintile 3	.016	−.023	.021
Quintile 4	.093***	.154***	−.052***
Quintile 5 (highest)	.242***	.255***	−.106***
Social participation index^d^	.178***	.197***	−.135***
FV intake (sufficient)	.185***	.153***	−.073***
PA (active)	.095***	.105***	−.197***
Daily smoker (yes)	.004	.025	−.034

*QoL* quality of life, *FV* fruit and vegetable, *PA* physical activity

***p < 0.001

^a^Higher scores represent better QoL

^b^Higher scores represent better cognitive function

^c^Higher scores represent poorer physical function

^d^Higher scores indicate more social participation

Table 3 demonstrates the associations of health behaviours and quality of life to health outcomes in analyses controlled for background characteristics. Social participation was associated significantly with all health and quality of life outcomes. With all other variables held constant, a 1-unit increase in the social participation index score was associated with a 0.128-unit increase in the quality of life score [β = 0.128, standard error (SE) = 0.002, *p* < 0.001], a 0.154-unit increase in the cognitive function score (β = 0.154, SE = 0.033, *p* < 0.001) and a 0.101-unit decrease in the physical function score (β = − 0.101, SE = 0.008, *p* < 0.001). Compared with insufficient intake, sufficient fruit and vegetable intake was associated with a 0.087-unit increase in the quality of life score (β = 0.087, SE = 0.015, *p* < 0.001). Compared with physical inactivity, physical activity was associated with a 0.091-unit increase in the quality of life score (β = 0.091, SE = 0.015, *p* < 0.001) and a 0.155-unit decrease in the physical function score (β = − 0.155, SE = 0.062, *p* < 0.001). No significant association was found between daily smoking and any health outcome or the quality of life score (Table 3).

**Table 3:** Multivariate regression results for relationships of health behaviours to QoL and health outcomes. Analyses were controlled for background characteristics.

	QoL^a^				Cognitive function^b^				Physical function^c^			
	Unstandardized coefficients		Standardized coefficients	*p*	Unstandardized coefficients		Standardized coefficients	*p*	Unstandardized coefficients		Standardized coefficients	*p*
	B	SE	Beta		B	SE	Beta		B	SE	Beta	
Age (years)	.003	.001	.048	<.001	−.247	.014	−.224	<.001	.047	.004	.179	<.001
Gender (female)	−.035	.016	−.030	.031	−1.544	.270	−.076	<.001	.065	.069	.013	.344
Residence (rural)	.003	.017	.003	.849	−3.153	.271	−.154	<.001	.595	.069	.122	<.001
Marital status (non-single)	.073	.019	.048	<.001	.838	.318	.032	.008	−.118	.081	−.019	.147
Education (lower)	−.065	.017	−.054	<.001	−3.358	.274	−.162	<.001	.0.18	.070	.004	.797
Income (quintile 2)	.154	.023	.103	<.001	.809	.371	.032	.029	−.293	.095	−.048	.002
Income (quintile 3)	.282	.023	.193	<.001	2.092	.375	.083	<.001	−.164	.095	−.028	.085
Income (quintile 4)	.350	.023	.246	<.001	4.771	.378	.195	<.001	−.438	.096	−.075	<.001
Income (quintile 5, highest)	.507	.024	.354	<.001	5.897	.399	.234	<.001	−.581	.101	−.098	<.001
**Health behaviours**												
Social participation index^d^	.021	.002	.128	<.001	.437	.033	.154	<.001	−.068	.008	−.101	<.001
FV intake (sufficient)	.107	.015	.087	<.001	.267	.248	.013	.282	−.032	.064	−.006	.618
PA (active)	.112	.015	.091	<.001	.718	.245	.033	.003	−.786	.062	−.155	<.001
Daily smoker (yes)	.008	.020	.006	.689	−.536	.325	−.021	.100	−.071	.083	−.012	.393
Constant	2.586	.076	–	<.001	48.880	1.249	–	<.001	−.467	.318	–	.142
Overall adjusted *R*^2^	.160				.293				.113			
Model *F* value	90.38			<.001	185.09			<.001	61.87			<.001
*n*	6099				5761				6200			

*QoL* quality of life, *SE* standard error, *FV* fruit and vegetable, *PA* physical activity

^a^Higher scores represent better QoL

^b^Higher scores represent better cognitive function

^c^Higher scores represent poorer physical function

^d^Higher scores represent more social participation

## Discussion

Previous studies have linked social participation to various quality of life and health outcomes among older adults [20, 21], but not specifically among chronically ill older adults. Moreover, they did not involve the investigation of social participation as a health behaviour in addition to traditional health behaviours (i.e. physical activity, maintenance of a healthy diet and not smoking). In this study, we thus examined the associations of social participation and traditional health behaviours with quality of life and health outcomes among chronically ill older people in China.

We found that the health behaviour social participation was associated significantly with all health and quality of life outcomes examined, which was not the case for traditional health behaviours (smoking, healthy diet, and physical activity). Among all health behaviours, social participation showed the strongest association with better quality of life. In contrast, Hu and colleagues [29] failed to find an association between social participation and quality of life among older Chinese adults with type 2 diabetes. However, they focused mainly on participation in formal organisations, such as sports clubs, which is not common among older Chinese adults and may have contributed to the lack of association [29]. In the current study, we incorporated broader aspects of social participation (e.g. working with other neighbourhood residents to fix or improve something and participation in social events in other neighbourhoods), which are more common among older Chinese adults. Our findings extend our understanding of the importance of social participation as an additional health behaviour in chronically ill older populations. Health promotion and lifestyle programmes for such populations should thus address social participation as well as traditional health behaviours.

Physical activity was not associated with cognitive function in our study, in contrast to the previous finding of a positive association among older adults with hypertension [16]. In an intervention study conducted with diabetic patients [54], physical activity was related to certain aspects of cognitive function, such as memory and executive function, but was not associated with other aspects (i.e. psychomotor speed and attention/concentration). The inconsistency among findings may reflect the use of different measures of cognitive function. For instance, Frith and Loprinzi [16] used the digit symbol substitution test, whereas we used a more comprehensive measure of cognitive function. Wu et al.’s [54] study might partly explain the lack of association in our study because our measure of cognitive function incorporated aspects of attention and concentration, which were shown to be unrelated to physical activity.

In the present study, we observed no association between smoking and any health or quality of life outcome examined in the bivariate correlation and multivariate regression analyses. Similarly, no association has been reported among patients with diabetes [55, 56] and hypertension [57]. Nevertheless, in general, smoking has been associated with decreased quality of life among chronically ill patients, including those with diabetes, asthma and lung cancer [58–60]. The reason for the lack of association in our study remains unknown. Research has suggested that smoking intensity (i.e. years of smoking, number of cigarettes per day) influences associations between smoking and health outcomes [61, 62]. However, most reports do not provide information on smoking intensity, and smoking status has been classified in different ways, making comparison among studies difficult. For example, Xu and colleagues [57] dichotomized smoking status (‘smoking’ and ‘no smoking’), Danson et al. [60] used three categories (never, former and current smokers) and we used the most commonly employed dichotomized variable (‘daily smoker’ and ‘not a daily smoker’). Differences in controlling for confounders among studies also may have contributed to the variation in associations [62]. For example, Danson et al. [60] study controlled for demographic and clinical variables (e.g. long-term health problems and previous medical conditions), whereas Cataldo et al. [63] controlled only for age, gender and depression. In addition, the higher mortality rate of heavy smokers may have biased the analyses [64].

### Study strengths and limitations

Our study has several strengths. First, it demonstrated that traditional health behaviours and social participation influenced quality of life and health outcomes in a large nationally representative sample of chronically ill older adults in China. Second, to minimise confounding bias, we included various potential confounders (e.g. socio-demographic characteristics) in the regression model. Third, although we could not assess causality, our findings show that chronically ill older adults may benefit from social participation.

Nevertheless, our findings should be viewed in light of the study’s limitations. As this study was the first to investigate health behaviours of social participation, smoking, physical activity and maintenance of a healthy diet simultaneously with health and quality of life outcomes among chronically ill older adults in China, more research is needed to support our study findings and increase their generalisability. Second, although we followed the WHO’s guideline in defining a healthy diet by measuring fruit and vegetable intake, this measure might be too general, which may have influenced the associations in our analysis. More research is needed to confirm associations with more inclusive dietary criteria, such as those for meat, dairy products, eggs, fish, poultry and soybeans, which are more commonly consumed in China [65]. Future research also should consider the impacts of the consumption of (certain amounts) of unhealthy foods, such as fatty and high-calorie foods [66]; diets including large amounts of unhealthy foods should not be considered to be healthy, even when they also include sufficient amounts of fruits and vegetables. Third, due to the cross-sectional design of this study, we could not examine the causality of associations of social participation and health behaviours with quality of life and health outcomes. Social participation and physical function may be reciprocally related [67]. Future studies should investigate whether changes in social participation and health behaviours are associated with improvements in quality of life and health outcomes among chronically ill patients over time; the effects of changes in health and quality of life outcomes on social participation and health behaviours should also be explored. Finally, we do not know whether or how chronic condition severity and combinations affect health behaviours and health outcomes due to data limitations. Research has suggested that hypertension, chronic hyperglycaemia and atherosclerotic macrovascular disease have a combined effect on cognitive function in patients with type 2 diabetes [56]. Future studies should consider the potential combined effects of multiple chronic diseases, as multimorbidity is common in older adults.

## Conclusions

This study showed that social participation is an important health behaviour for health and quality of life outcomes among chronically ill older adults in China. Expansion of the focus of health promotion programmes and lifestyle interventions to include social participation as an additional health behaviour is thus expected to be beneficial.

## Supplementary information

**Additional file 1.** Social participation index.

**Additional file 2.** Eight-item World Health Organization quality of life measure (WHOQoL).

## Data Availability

The datasets analysed for the current study are available in the World Health Organization’s Multi-Country Studies Data Archive repository (http://apps.who.int/healthinfo/systems/surveydata/index.php/catalog).

## Abbreviations

CAPI: Computer-assisted personal interviewing
HRS: US Health and Retirement Study
KLoSA: Korean Longitudinal Study on Ageing
SAGE: Study on Global AGEing and Adult Health
SE: standard error
WHO: World Health Organization
WHOQoL: World Health Organization Quality of Life measure
