# Supplementary material for: Social participation is an important health behaviour for health and quality of life among chronically ill older Chinese people
Source: BMC Geriatr. 2020 Aug 24;20:299. doi: 10.1186/s12877-020-01713-6 (PMC7444063; doi:10.1186/s12877-020-01713-6)
Supplement: Supplementary file 1 — Additional file 1. Social participation index. [file 12877_2020_1713_MOESM1_ESM.docx]

**Appendix 1**

**Social participation index**

How often in the last 12 months have you…

| 1. Attended any public meeting in which there was a discussion of local or school affairs?  2. Met personally with someone you consider to be a community leader?  3. Attended any group, club, society, union or organisational meeting?  4. Worked with other people in your neighbourhood to fix or improve something?  5. Had friends over to your home?  6. Been in the home of someone who lives in a different neighbourhood than you do or had them in your home?  7. Socialised with co-workers outside of work?  8. Attended religious services (not including weddings and funerals)?  9. Gotten out of the house/your dwelling to attend social meetings, activities, programmes or events or to visit friends or relatives? |
| --- |
