# Supplementary material for: Social participation is an important health behaviour for health and quality of life among chronically ill older Chinese people
Source: BMC Geriatr. 2020 Aug 24;20:299. doi: 10.1186/s12877-020-01713-6 (PMC7444063; doi:10.1186/s12877-020-01713-6)
Supplement: Supplementary file 2 — Additional file 2. Eight-item World Health Organization quality of life measure (WHOQoL). [file 12877_2020_1713_MOESM2_ESM.docx]

***Appendix 2***

***Eight-item World Health Organization quality of life measure (WHOQoL)***

| 1 | Do you have enough energy for everyday life? |
| --- | --- |
| 2 | Have you enough money to meet your needs? |
| 3 | How satisfied are you with your health? |
| 4 | How satisfied are you with your ability to perform your daily living activities? |
| 5 | How satisfied are you with your personal relationships? |
| 6 | How satisfied are you with the conditions of your living place? |
| 7 | How satisfied are you with your life as whole these days? |
| 8 | How would you rate your quality of life? |
